# *ACIP Updates:* Recommendations for Use of 20-Valent Pneumococcal Conjugate Vaccine in Children ― United States, 2023

**DOI:** 10.15585/mmwr.mm7239a5

**Published:** 2023-09-29

On June 22, 2023, the Advisory Committee on Immunization Practices (ACIP) convened and approved recommendations for the use of 20-valent pneumococcal conjugate vaccine (PCV20 [Prevnar 20; Wyeth Pharmaceuticals, Inc., a subsidiary of Pfizer, Inc.]) in U.S. children. The ACIP recommendations were adopted by the CDC Director on June 27, 2023, and are official. The recommendations, underlying evidence and rationale, and clinical guidance are available (Supplementary Report, https://stacks.cdc.gov/view/cdc/133252).

